# Supplementary material for: The Development of Human Ex Vivo Models of Inflammatory Skin Conditions
Source: Int J Mol Sci. 2023 Dec 8;24(24):17255. doi: 10.3390/ijms242417255 (PMC10743306; doi:10.3390/ijms242417255)
Supplement: Supplementary file 1 [file ijms-24-17255-s001.zip › ijms-2750807-supplementary.pdf]

Supplementary Materials

Supplementary Table 1. Antibodies used in immunohistochemistry

Antibody	Clone	Vendor	HIAR	Dilution
Anti-CD3	SP7	Abcam; Ab16669	pH 6	1:100
Anti-CD45	EP322Y	Abcam; Ab40763	pH 9	1:100
Anti-CD68	EPR20545	Abcam; Ab213363	pH 9	1:600
Anti-Filaggrin	FLG01	ThermoFisher; MA5-13440	pH 9	1:400
Anti-Loricrin	Polyclonal	Abcam; Ab85679	pH 6	1:250

Supplementary Table 2. Donor demographics

Ethnicity	Age	Sex	Models Used
African American	54	Female	AG, IR, CS
Hispanic	60	Female	AG, CS
Hispanic	26	Female	CS
Hispanic	63	Female	AG, IR
Caucasian	45	Female	AG, IR
Caucasian	63	Female	IR, CS
